# Implication of Ceramide Kinase in Adipogenesis

**DOI:** 10.1155/2017/9374563

**Published:** 2017-07-20

**Authors:** Marta Ordoñez, Natalia Presa, Miguel Trueba, Antonio Gomez-Muñoz

**Affiliations:** Department of Biochemistry and Molecular Biology, Faculty of Science and Technology, University of the Basque Country (UPV/EHU), 48080 Bilbao, Spain

## Abstract

Ceramide kinase (CerK) plays a critical role in the regulation of cell growth and survival and has been implicated in proinflammatory responses. In this work, we demonstrate that CerK regulates adipocyte differentiation, a process associated with obesity, which causes chronic low-grade inflammation. CerK was upregulated during differentiation of 3T3-L1 preadipocytes into mature adipocytes. Noteworthy, knockdown of CerK using specific siRNA to silence the gene encoding this kinase resulted in substantial decrease of lipid droplet formation and potent depletion in the content of triacylglycerols in the adipocytes. Additionally, CerK knockdown caused blockade of leptin secretion, an adipokine that is crucial for regulation of energy balance in the organism and that is increased in the obese state. Moreover, CerK gene silencing decreased the expression of peroxisome proliferator-activated receptor gamma (PPAR*γ*), which is considered the master regulator of adipogenesis. It can be concluded that CerK is a novel regulator of adipogenesis, an action that may have potential implications in the development of obesity, and that targeting this kinase may be beneficial for treatment of obesity-associated diseases.

## 1. Introduction

Adipogenesis is the development of fully differentiated mature adipocytes from precursor cells. Apart from its role as triacylglycerol (TG) storage and energy homeostasis, adipose tissue is an endocrine organ that regulates crucial pathophysiological processes. Classical adipocyte-specific hormones include leptin [1], adiponectin [2], or resistin [3], but preadipocytes and mature adipocytes can also secrete numerous pro- and anti-inflammatory cytokines. Many of these so-called adipokines are involved in the regulation of glucose and lipid metabolism and are implicated in conditions of insulin resistance and obesity [4–6].

In recent years, sphingolipids and sphingolipid-metabolizing enzymes have emerged as key regulators of vital cellular functions including cell growth, differentiation, or cell death, and some of them are involved in inflammatory responses and inflammation-associated diseases such as cardiovascular diseases, cancer, and obesity. In particular, ceramides have been associated with insulin resistance leading to type II diabetes, and sphingosine and sphingosine kinases are altered in the obese state [7, 8]. However, a putative role for ceramide kinase (CerK), the enzyme that phosphorylates ceramide to produce ceramide 1-phosphate (C1P), in adipogenesis has remained largely unknown.

The present study was undertaken to evaluate whether CerK was implicated in adipocyte differentiation, which is a target for therapy of obesity-related diseases.

## 2. Materials and Methods

### 2.1. Materials

The culture medium Dulbecco's modified Eagle's medium (DMEM) used in all the experiments was purchased from Lonza. The triacylglycerol assay kit was purchased from Abnova. NBD-ceramide (NBD-N-hexanoyl-D-erythro-sphingosine) was from Cayman Chemicals. Dexamethasone, rosiglitazone, insulin, 3-isobutyl-1-methylxanthine (IBMX), and the Oil Red O dye were obtained from Sigma-Aldrich. Nitrocellulose membranes, protein markers, and BCA assay reagents were purchased from Bio-Rad. Fetal bovine serum (FBS) and newborn calf serum (NCS) were from GIBCO. The ELISA kit for determination of leptin was purchased from Peprotech. The PPAR*γ* antibody was supplied by Cell Signaling. The GAPDH antibody, nontargeting (negative) siRNA, and ceramide kinase (CerK) siRNA were purchased from Santa Cruz Biotechnology. The CerK antibody was from Calbiochem or Abgent. The rest of chemicals and reagents used in this work were of the highest grade available.

### 2.2. Cell Culture

The 3T3-L1 cell line is a fibroblast cell line purchased from the American Type Culture Collection (ATCC) (Manassas, VA, USA) and was cultured following the manufacturer's indications. Cells were grown in 175 cm^2^ flasks in DMEM supplemented with 10% heat-inactivated newborn calf serum (NCS), 50 mg/l gentamicin, 200 *μ*M L-glutamine, and 4.5 g/l glucose. Cells were incubated in a humidified 5% CO_2_ incubator at 37°C and subcultured every 3-4 days. Cells were used in experiments at about 80–100% confluence.

### 2.3. 3T3-L1 Preadipocyte Differentiation Protocol

3T3-L1 preadipocytes were seeded in 6-well plates at 1.2 × 10^5^ cells/well or in 24-well plates at 6 × 10^4^ cells/well or in 96-well plates at 9 × 10^3^ cells/well depending on the kind of experiment to be performed. The cells were then cultured in DMEM supplemented with 10% NCS until they were about 90–100% confluent. The cells were then further incubated for 2 days. The preadipocytes were treated with adipogenic induction medium, which is a medium consisting of DMEM 10% FBS supplemented with an adipogenic cocktail containing 0.5 mM IBMX, 1 *μ*g/ml insulin, 0.25 *μ*M dexamethasone, and 2 *μ*M rosiglitazone. Two days later, the medium was removed and cells were incubated further in maintenance medium (DMEM 10% FBS + 1 *μ*g/ml insulin) for two additional days. The cells were then fed every two days with DMEM supplemented with 10% FBS and 1 *μ*g/ml insulin.

### 2.4. Western Blotting

Preadipocytes were harvested and lysed in ice-cold homogenization buffer to analyze proteins by Western blotting, essentially as described in [9]. Specifically, 3T3-L1 cells were seeded at 1.2 × 10^5^ cells/well in 6-well plates and were differentiated in the presence or in the absence of CerK siRNA, following the above described preadipocyte differentiation protocol (Section 2.3). Western blotting was performed as detailed in [10]. About 20–40 *μ*g of protein from each sample was loaded and separated by sodium dodecyl sulfate polyacrylamide gel electrophoresis (SDS-PAGE), using 12% separating gels. Proteins were transferred onto nitrocellulose membranes and blocked with 5% skim milk for 1 h in Tris-buffered saline (TBS) containing 0.1% Tween 20 and then incubated overnight with the primary antibody in TBS-0.1% Tween 20 at 4°C. After three washes with TBS-0.1% Tween 20, membranes were incubated with horseradish peroxidase-conjugated secondary antibody at 1 : 4000 dilution for 1 h. Bands were visualized by enhanced chemiluminescence and exposed films were analyzed with an ImageJ software.

### 2.5. Oil Red O Staining Protocol

3T3-L1 preadipocytes were seeded at 6 × 10^4^ cells/well in 24-well plates and differentiated in the absence or in the presence of CerK siRNA, following the above described preadipocyte differentiation protocol (Section 2.3). Intracellular lipid accumulation was determined using a solution of Oil Red O. Briefly, cells were washed with PBS and fixed in 3.8% formaldehyde for 10 min. They were then washed and stained with a solution containing Oil Red O (3 mg/ml) and isopropanol in water (60/40, *v*/*v*) for 20 min at room temperature. Stained cells were washed twice with water and photographed with a Nikon Eclipse TS100 microscope. To assess the degree of differentiation, 200 *μ*l isopropanol was added and incubated for 30 min in a plate shaker. Then, 50 *μ*l of Oil Red O extracted dye was transferred into 96-well plates and quantified by reading the absorbance at a wavelength of 510 nm. The dye extracted from the empty wells represented the nonspecific binding of the dye to the plate. The nonspecific binding value was subtracted from the absorbance of each experimental condition to obtain accurate measurements of specific staining.

### 2.6. Triacylglycerol Measurement

3T3-L1 preadipocytes were seeded at 9 × 10^3^ cells/well in 96-well plates and differentiated in the absence or in the presence of CerK siRNA, following the above described preadipocyte differentiation protocol (Section 2.3). For determination of the triglyceride (TG) content in cells, the manufacturer's instructions were followed. Briefly, cells were washed with PBS and 100 *μ*l of the lipid extraction solution was added to each well. Then, the plates were incubated in a heating block at 90–100°C for 30 min. After this time, 50 *μ*l/well of standard dilutions of TG or 5–50 *μ*l of the lipid extracts were transferred into 96-well plates. Assay buffer was added where necessary to bring the volume up to 50 *μ*l in all wells. Then, 2 *μ*l of lipase solution was added to each well containing either sample or standard, and the whole plate was incubated for 10 min at room temperature. Subsequently, 50 *μ*l of the reaction mixture (46 *μ*l adipogenesis assay buffer + 2 *μ*l probe + 2 *μ*l enzyme mix) was added to each well and incubated at 37°C for 30 min in the dark. The absorbance was read at 570 nm in a plate reader. The protein concentration of the lipid extracts was determined using a commercial kit containing bicinchonic acid (BCA), from Bio-Rad, and the values were used as internal controls to normalize the lipid concentration in the samples.

### 2.7. Treatment of Cells with siRNA

3T3-L1 preadipocytes were seeded in 100 mm diameter dishes at 5 × 10^5^ cells/plate and incubated for 48 h as indicated in Section 2.2. The cells were then transferred into electroporation cuvettes and 20 pmol/ml of siRNA (from a 10 *μ*M siRNA stock solution) was added. Cells were then electroporated (1000 v, 30 *μ*S) using an Electro Square Porator (ECM 830). Electroporated cells were then seeded at 2 × 10^4^ cells/well in 96-well plates or 1.2 × 10^5^ cells/well in 24-well plates or 5 × 10^5^ cells/well in 6-well plates, depending on the particular experiment to be performed, and incubated in differentiation medium, as required. The silencing efficiency of the siRNA treatment was analyzed by Western blotting and by measuring CerK activity.

### 2.8. Determination of CerK Activity

CerK activity was essentially determined as described by Don and Rosen [11]. 3T3-L1 cells were seeded in 6-well plates at 1.2 × 10^5^ cells/well and differentiated in the absence or in the presence of CerK siRNA, following the preadipocyte differentiation protocol described above (Section 2.3). Cell lysates (50–100 *μ*g of protein) were mixed with reaction buffer (100 *μ*l, 20 mM Hepes (pH 7.4), 10 mM KCl, 15 mM MgCl2, 15 mM CaCl_2_, 10% glycerol, 1 mM DTT, and 1 mM ATP) containing 10 *μ*M of NBD-C6-ceramide (N-hexanoyl-D-erythro-sphingosine). The reactions were allowed to proceed for 30 min in the dark. Then, 250 *μ*l aliquots of chloroform : methanol (2 : 1) were added to terminate the reactions. Samples were then centrifuged at 280,800*g* for 30 s, and 100 *μ*l aliquots of the upper aqueous phase were transferred into 96-well plates. Subsequently, 100 *μ*l aliquots of dimethylformamide (DMF) were added before reading the NBD fluorescence in an appropriate plate reader. Fluorescence was quantified with a 495 nm excitation filter and a 520 nm emission filter with a Synergy HT (Biotek) plate reader equipped with Gen5 software.

### 2.9. Determination of C1P Levels

C1P was analyzed by labeling the cells with [^3^H]palmitate essentially as described in [12], followed by separation of C1P by thin-layer chromatography [13]. Specifically, 3T3-L1 preadipocytes were seeded in 6-well plates at 1.2 × 10^5^ cells/well and incubated in differentiation medium for four days, following the above described preadipocyte differentiation protocol (Section 2.3). [^3^H]Palmitate (2 *μ*Ci/ml) was present in the culture medium for the last 24 h. The radioactive medium was then removed and the cells were washed twice with nonradioactive DMEM and collected for determination of [^3^H]C1P formation. To this purpose, the cells were scraped in 0.5 ml of methanol, and the wells were washed with an equal volume of methanol. The two 0.5 ml methanol samples were combined and mixed with 0.5 ml of chloroform. The lipids were extracted by separation of phases with an additional volume of 0.5 ml of chloroform to which 0.9 ml of a solution containing 2 M KCl and 0.2 M HCl was added. The chloroform phases were isolated and dried down under a stream of nitrogen. Subsequently, glycerophospholipids were eliminated by mild alkaline hydrolysis incubating the samples in 0.5 M methanolic KOH at 37°C for 30 min with continuous shaking. After the hydrolysis, lipids were re-extracted by separation of phases as indicated above. The lipid extracts were dissolved in 50 *μ*l of chloroform containing 50 *μ*g of cold C1P as carrier and were applied directly to aluminum- or glass-backed silica gel 60 plates to separate the lipids. The silica gel plates were developed sequentially with three different solvent systems. Solvent 1 consisted of chloroform/methanol/NH_4_OH (65 : 35 : 7.5, *v*/*v*/*v*) and was run up to 11.5 cm from the origin, which was set up at 1.5 cm from the bottom of the plate. Solvent 2 contained chloroform/methanol/acetic acid (9 : 1 : 1, *v*/*v*/*v*) and was run up to the top of the plate. Solvent 3 consisted of 1-butanol/acetic acid/water (3 : 1 : 1, *v*/*v*/*v*) and was run up to 14 cm from the origin. The plates were dried in a fume hood after each solvent run before they were developed in the next corresponding solvent. C1P was identified after staining the plates with iodine vapor by comparison with authentic standards. Radioactivity was quantified by scraping the C1P spots from the silica plates by liquid scintillation counting.

### 2.10. Statistical Analyses

Results are expressed as means ± SEM of three to six independent experiments performed in duplicate unless indicated otherwise. Statistical analyses were performed using the two-tailed, paired Student's *t*-test, where *p* < 0.05 was considered to be significant (GraphPad Prism Software, San Diego, CA) [10].

## 3. Results

### 3.1. CerK Is Upregulated during Adipogenesis in 3T3-L1 Cells

**Figure 1:**
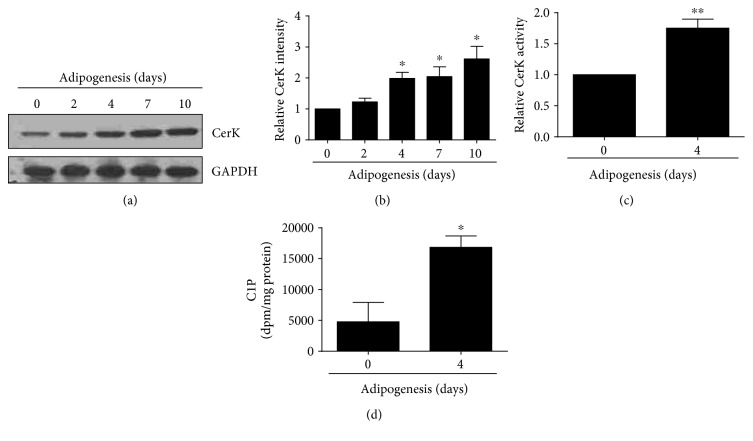
CerK is upregulated during adipogenesis. Cells were seeded in 6-well plates (1.2 × 10^5^ cells/well), and they were differentiated as described in Materials and Methods. (a) CerK was detected by Western blotting using a specific antibody. Equal loading of protein was assessed with an antibody against GAPDH. Similar results were obtained in each of 4 replicate experiments. (b) Results of the scanning densitometry of exposed film. Data are expressed as arbitrary units of intensity relative to GAPDH and are the mean ± SEM of 4 independent experiments (^∗^*p* < 0.05). (c) CerK activity was determined as described in Materials and Methods. Data are expressed as the mean ± SEM of 5 independent experiments (^∗∗^*p* < 0.01). (d) C1P levels were determined as described in Materials and Methods. Data are expressed as the mean ± SEM of four independent determinations (^∗^*P* < 0.05).

The implication of CerK in adipogenesis was tested using 3T3-L1 mouse preadipocytes. We observed that the protein level of CerK was upregulated under adipogenic differentiation conditions when the cells were incubated up to 10 days in differentiation cell culture medium (Figures 1(a) and 1(b)). We also observed that CerK activity increased significantly under differentiation conditions (Figure 1(c)) and that C1P levels were elevated by about 3.5-fold when the cells were preincubated with 2 *μ*Ci/ml of [^3^H]palmitate to label cell lipids, including sphingolipids, under the same conditions (Figure 1(d)).

### 3.2. CerK Contributes to Adipogenesis in 3T3-L1 Cells

**Figure 2:**
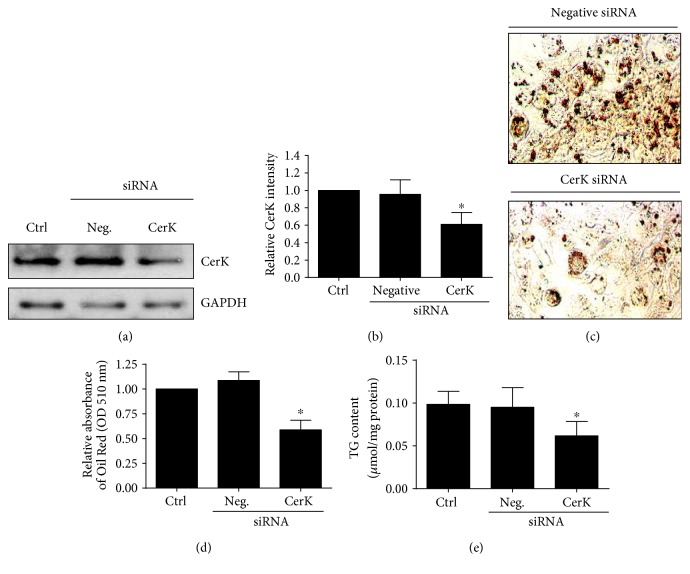
CerK downregulation leads to decreased lipid droplet formation and depletion of TG content in 3T3-L1 cells. The preadipocytes were seeded in 100 mm diameter dishes at 5 × 10^5^ cells/plate and incubated for 48 h as indicated in Section 2.2. They were then electroporated in the absence of siRNA (ctrl) or in the presence of negative (scrambled) siRNA (Neg siRNA) or Cerk siRNA, as indicated in Materials and Methods, and were then differentiated up to day 4. (a) CerK knockdown using specific siRNA was confirmed by immunoblotting. Equal loading of protein was assessed with an antibody against GAPDH. Similar results were obtained in each of 3 replicate experiments. (b) Results of the scanning densitometry of exposed film. Data are expressed as arbitrary units of intensity relative to GAPDH and are the mean ± SEM of 3 independent experiments (^∗^*p* < 0.05). (c) Light micrographs of adipocytes stained with Oil Red O at day 4 after differentiation are shown. Pictures were taken with a motorized Nikon Eclipse TS100 microscope at 20x magnification. (d) Cells were stained with Oil Red O, and lipid droplets were quantified at day 4 after differentiation as indicated in Materials and Methods. Data are expressed as the mean ± SEM of 5 independent experiments performed in triplicate (^∗^*p* < 0.05). (e) Triacylglycerol (TG) content of cells was measured at day 4 after differentiation as described in Materials and Methods. Results are the mean ± SEM of 3 independent experiments performed in triplicate (^∗^*p* < 0.05).

The role of CerK in adipogenesis was investigated by transfecting siRNA targeting CerK into 3T3-L1 preadipocytes to silence the gene encoding this kinase. The knockdown of CerK by siRNA treatment was confirmed by immunoblotting (Figures 2(a) and 2(b)). Surprisingly, cells deficient in CerK resulted in reduced adipogenic differentiation and decreased formation of lipid droplets compared to control cells. Lipid droplets were detected by Oil Red O staining at 4 days after induction of adipogenesis (Figures 2(c) and 2(d)). In addition, the content of TG was substantially decreased in siRNA CerK-treated cells compared to control cells (Figure 2(e)).

### 3.3. CerK Deficiency Results in Reduced Leptin Release and Inhibition of PPAR*γ* Expression

**Figure 3:**
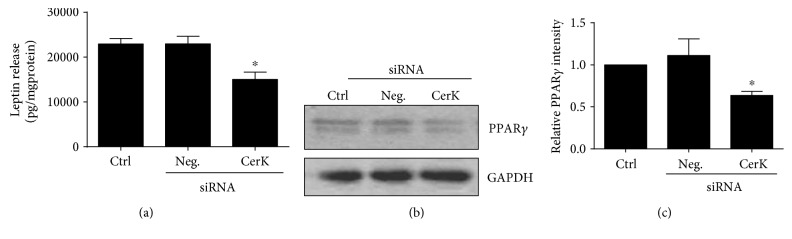
CERK downregulation leads to inhibition of leptin secretion and inhibition of PPAR-*γ* expression. The preadipocytes were seeded in 100 mm diameter dishes at 5 × 10^5^ cells/plate and incubated for 48 h as indicated in Section 2.2. The cells were then electroporated in the absence of siRNA (ctrl) or in the presence of negative (scrambled) siRNA (Neg siRNA) or Cerk siRNA, as indicated in Materials and Methods. (a) Leptin concentration in supernatants was measured at day 4 after differentiation using an ELISA kit, as described in Materials and Methods. Results are the mean ± SEM of 4 independent experiments performed in duplicate (^∗^*p* < 0.05). (b) PPAR*γ* expression was detected by Western blotting using a specific antibody. Equal loading of protein was assessed with an antibody against GAPDH. Similar results were obtained in each of 5 replicate experiments. (c) Results of scanning densitometry of the exposed film. Data are expressed as arbitrary units of intensity relative to GAPDH and are the mean ± SEM of 5 independent experiments (^∗^*p* < 0.05).

Leptin is the central regulator of energy balance and appetite in the organism. It is produced only after the induction of adipocyte differentiation, and as such, it has been used as a late marker of adipogenesis [14]. In addition, leptin exerts proinflammatory actions in the obese state [14]. We show in this work that CerK knockdown with specific CerK siRNA significantly decreases leptin release by the adipocytes during cell differentiation (Figure 3(a)), thereby reinforcing the notion that CerK is a relevant factor in adipogenesis.

Many proadipogenic transcription factors have been characterized, but no one is as critical as the peroxisome proliferator-activated receptor gamma (PPAR*γ*), which is considered the master regulator of adipocyte differentiation [4]. In fact, the majority of factors controlling adipocyte differentiation also affect the activity of this crucial regulator of adipogenesis [15]. Noteworthy, we show in this work that CerK knockdown with specific CerK siRNA significantly decreases PPAR*γ* protein levels in the adipocytes during cell differentiation (Figures 3(b) and 3(c)), suggesting that the expression of this transcription factor is under regulation by CerK.

## 4. Discussion

In the present study, we have investigated the role of CerK in adipogenesis using 3T3-L1 preadipocytes as cellular model. CerK expression was increased during adipogenic differentiation, and CerK knockdown resulted in impaired adipogenesis as determined by the reduction of lipid droplet formation and the TG content of cells. The latter results are consistent with recent work showing that CerK deficiency improves diet-induced obesity and insulin resistance [16]. CerK deficiency resulted in significant decrease of leptin secretion, an adipokine that is increased in the obese state [4, 14]. Leptin has proinflammatory properties and can induce the secretion of inflammatory cytokines such as tumor necrosis factor-alpha (TNF-*α*), interleukin-6 (IL-6), or IL-12 [17, 18]. In turn, proinflammatory TNF-*α* and IL-1*β* can upregulate the expression of leptin mRNA in the adipose tissue, leading to a loop that potentiates inflammation [18, 19]. Therefore, the reduction of leptin levels by downregulation of CerK would attenuate the proinflammatory effects of this adipokine. This is an important aspect in the context of obesity, which is a low-grade inflammatory condition. The involvement of CerK in inflammation was first suggested by Chalfant and coworkers who showed that CerK mediates IL-1*β*-induced arachidonic acid release, which is the precursor of inflammatory eicosanoids [20]. Subsequent studies by the same group demonstrated that C1P, the product of CerK, was a direct activator of cytosolic phospholipase A_2_ [21], a major enzyme responsible for the regulation of arachidonic acid release in mammalian cells, and that eicosanoid levels were lower in primary mouse embryonic fibroblasts isolated from CerK knockout mice [22]. Moreover, depleting CerK activity using either specific siRNA to silence the gene encoding this kinase or the pharmacological inhibitor NVP-231 completely blocked eicosanoid biosynthesis [23]. Additionally, we show here that CerK-deficient cells cause potent reduction in the protein levels of the crucial adipogenesis marker PPAR*γ*, which is both necessary and sufficient for adipocyte differentiation [24], thereby suggesting that the loss of CerK might be detrimental for optimal adipogenic differentiation.

The above results suggest that CerK activity might regulate adipocyte differentiation by targeting the expression of adipogenic genes. In addition, the increased expression of CerK and C1P formation during adipogenesis are consistent with the substantial reduction of ceramide levels during differentiation of 3T3-L1 preadipocytes [25], as CerK produces C1P from ceramides. Although this may be a major mechanism for controlling adipogenesis, further investigation is required to identify the signaling pathways involved in this process and to clearly understand the molecular mechanisms that are implicated in CerK-mediated adipogenesis.

In summary, we have identified CerK as a novel regulator of adipogenesis. The increased levels of CerK expression during adipocyte differentiation point to a putative role of this kinase in the onset or development of obesity. In fact, obesity is an inflammatory condition that is primarily attributed to expansion and inflammation of adipose tissue [26]. It can be concluded that CerK is a new regulator of adipogenesis with potential implications in obesity and that targeting CerK may prove useful in the treatment of obesity-associated diseases.

## Abbreviations

BCA: Bicinchonic acid
C1P: Ceramide 1-phosphate
CerK: Ceramide kinase
DMEM: Dulbecco's modified Eagle's culture medium
FBS: Fetal bovine serum
IBMX: 3-Isobutyl-1-methyxanthine
PBS: Phosphate-buffered saline
PPAR*γ:*: Peroxisome proliferator-activated receptor gamma
TG: Triacylglycerol.
